# Cardiac Complications of Propionic and Other Inherited Organic Acidemias

**DOI:** 10.3389/fcvm.2020.617451

**Published:** 2020-12-22

**Authors:** Kyung Chan Park, Steve Krywawych, Eva Richard, Lourdes R. Desviat, Pawel Swietach

**Affiliations:** ^1^Department of Anatomy, Physiology and Genetics, Burdon Sanderson Cardiac Science Centre, British Heart Foundation Centre of Research Excellence, University of Oxford, Oxford, United Kingdom; ^2^Department of Chemical Pathology, Great Ormond Street Hospital, London, United Kingdom; ^3^Centro de Biología Molecular Severo Ochoa, Universidad Autonoma de Madrid-Consejo Superior de Investigaciones Cientificas (UAM-CSIC), Centro de Investigacion Biomedica en Red de Enfermedades Raras (CIBERER), IdiPaz, Universidad Autónoma de Madrid, Madrid, Spain

**Keywords:** inherited metabolic disease, metabolic acidosis, pH, organic anions, propionate, cardiomyopathy, dilated cardiomyopathy, arrhythmia

## Abstract

Clinical observations and experimental studies have determined that systemic acid-base disturbances can profoundly affect the heart. A wealth of information is available on the effects of altered pH on cardiac function but, by comparison, much less is known about the actions of the organic anions that accumulate alongside H^+^ ions in acidosis. In the blood and other body fluids, these organic chemical species can collectively reach concentrations of several millimolar in severe metabolic acidoses, as in the case of inherited organic acidemias, and exert powerful biological actions on the heart that are not intuitive to predict. Indeed, cardiac pathologies, such as cardiomyopathy and arrhythmia, are frequently reported in organic acidemia patients, but the underlying pathophysiological mechanisms are not well established. Research efforts in the area of organic anion physiology have increased dramatically in recent years, particularly for propionate, which accumulates in propionic acidemia, one of the commonest organic acidemias characterized by a high incidence of cardiac disease. This *Review* provides a comprehensive historical overview of all known organic acidemias that feature cardiac complications and a state-of-the-art overview of the cardiac sequelae observed in propionic acidemia. The article identifies the most promising candidates for molecular mechanisms that become aberrantly engaged by propionate anions (and its metabolites), and discusses how these may result in cardiac derangements in propionic acidemia. Key clinical and experimental findings are considered in the context of potential therapies in the near future.

## Introduction

Inherited metabolic disorders (IMDs), classically known as inborn errors of metabolism, are a collection of over 500 diseases, each caused by an inherited defect in genes coding for a metabolic enzyme. Although individually rare, as a collective they affect 1 in 2,000 births worldwide (1). IMDs exist for essentially every metabolic pathway, from fatty acid oxidation, to the urea cycle, and a number of them can be detected during newborn screening by tandem mass spectrometry (MS/MS). Recently, the role of whole-exome sequencing was evaluated as an innovative methodology to aid in the diagnosis of infants with abnormal MS/MS screens (2). Organic acidemias (OAs), sometimes referred to as organic acidurias, are a subset of 17 IMDs that are characterized by the excessive accumulation of organic acids in body fluids. OAs have been divided into three categories: (1) systemic, (2) cerebral, and (3) ketolytic/ketogenic (3), although this naming system is problematic because changes related to the brain are common in all OAs and the canonical cerebral OAs can also develop non-neurologic manifestations (4). With the advent of chemical assays capable of defining the metabolite milieu of blood, OAs have been related to specific metabolic signatures, some of which have diagnostic value. Modern genetic techniques have described the gene mutations that give rise to the metabolite disorder, and even provided some degree of insight into gene-function relationships.

A feature of many OAs is the accumulation of organic acid metabolites that directly relate to the mutated enzyme, typically accompanied by a build-up of various other acidic metabolites particularly during stages of metabolic decompensation, such as lactate, 3-hydroxybutyrate or acetoacetate. The clinical management of OAs is broadly based on the same principles, namely, the reversal or prevention of catabolism, limiting exposure to precursors of the substrate that cannot be metabolized, and scavenging toxic intermediates (3).

OAs are severe diseases that can affect multiple organ systems. Many OAs present in the neonatal or infantile period with a wide anion gap, metabolic acidosis and hyperammonemia. However, of the 17 known OAs, around half are known to cause cardiac dysfunction (Organic Acidemia Association, 2020). The disorder most strongly associated with cardiac sequelae is the life-threatening disease, propionic acidemia (PA), first reported by Dr. Barton Childs in 1961. PA, a relatively frequent type of OA, is characterized by an accumulation of propionate and other related metabolites, and is a systemic disorder resulting in widespread end-organ damage (5). Acute illness is typically a result of metabolic decompensation and acidosis. A common complication in PA is cardiomyopathy and arrhythmia, and while the underlying mechanisms are not yet fully established, notable achievements have been made recently (6–8). Methylmalonic acidemia (MMA), a condition closely related to PA, is also recognized to produce cardiac dysfunction, albeit not as frequently as in the case with PA. Similarly, there have been documented cases of heart disease in glutaric aciduria type II (multiple acyl-CoA dehydrogenase deficiency) and type II D-2-hydroxyglutaric aciduria.

**Figure 1:**
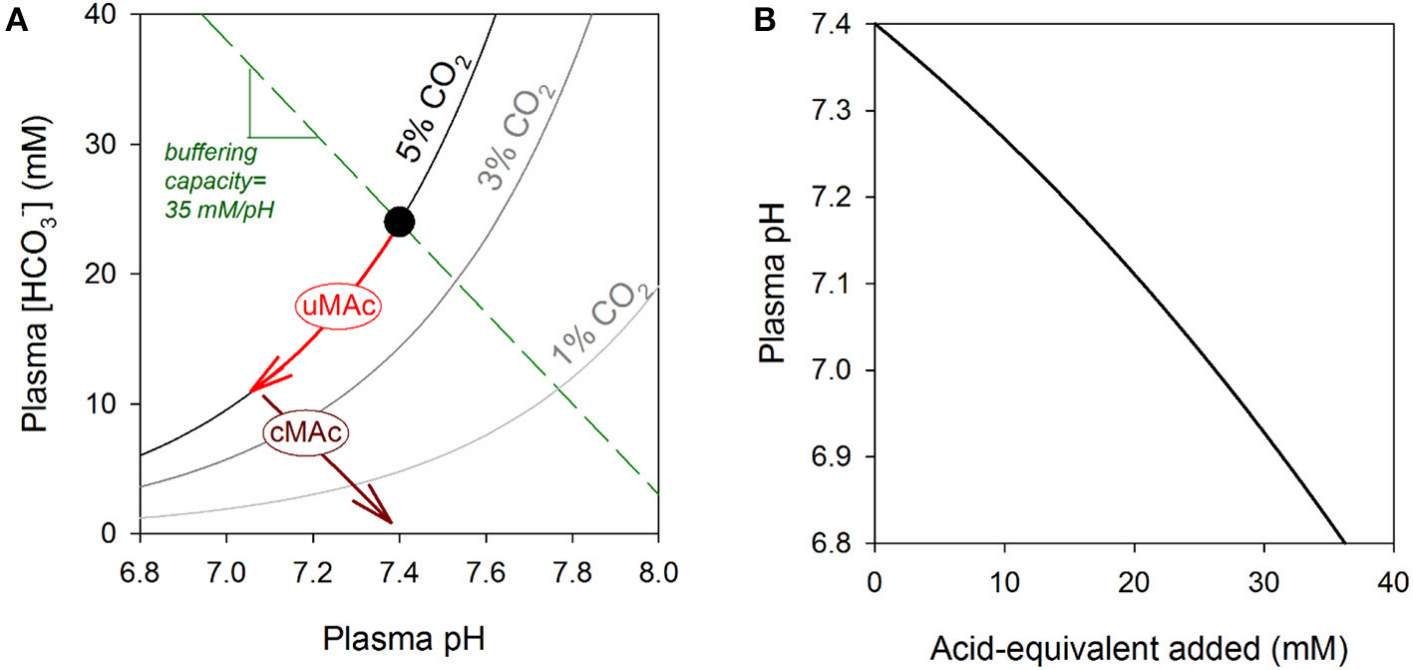
**(A)** Davenport diagram. Relationship between plasma pH and plasma [HCO${}_{3}^{-}$] shown for three CO_2_ isobars, including 5% (physiological) and two levels associated with hyperventilation (3 and 1%). Normal acid-base balance is represented by the black dot. A metabolic acidosis will result in a decrease in [HCO${}_{3}^{-}$]; if this is associated with a fall in pH, it is known as an uncompensated metabolic acidosis (uMAc) and its trajectory is along the 5% CO_2_ isobar. This metabolic acidosis can be compensated (cMAc) by hyperventilation to reduce CO_2_ and thus restore the [HCO${}_{3}^{-}$]/CO_2_ ratio to a level that is closer to pH 7.4. The direction of change will be parallel to the buffering capacity line, which is defined by the sum of all non-CO_2_/HCO${}_{3}^{-}$ buffers. For normal hematocrit, the buffering line has a slope close to 35 mM/pH. **(B)** Effect of adding non-volatile acid-equivalents on plasma pH. The gradient of this relationship is defined by carbonic and non-carbonic buffers. During a metabolic acidosis, even small changes in pH can be related to major disturbances in the levels of organic anions, the conjugate bases of metabolic acids. Change in pH was calculated as amount of acid added divided by buffering capacity. Buffering capacity due to non-carbonic sources was 35 mM/pH, and attributable mainly to hemoglobin in red cells. Buffering capacity due to CO_2_/HCO${}_{3}^{-}$ was calculated assuming constancy of CO_2_ at 5% (i.e., 2.303×[HCO${}_{3}^{-}$]), as expected for an open system. The calculation assumes a 0.2 pH gradient between plasma and red blood cell cytoplasm, and a hematocrit of 45%.

As their name implies, OAs have two distinct chemical components: H^+^ ions (which determine pH) and the conjugate organic anions. In aqueous solution, these ionic species are, in essence, fully dissociated, and can act via separate mechanisms. The maintenance of a favorable pH is a homeostatic priority, which is manifested by the observation that plasma pH is regulated within a narrow range of 7.35 to 7.45. The principal pH buffer present in plasma is CO_2_/HCO${}_{3}^{-}$, therefore the concentration of H^+^ ions, HCO${}_{3}^{-}$ ions and CO_2_ gas is in a dynamic equilibrium. Acid-base disturbances can emerge from a change in [HCO${}_{3}^{-}$] at constant partial pressure of CO_2_ (pCO_2_), referred to as a metabolic acidosis (fall in [HCO${}_{3}^{-}$]) or metabolic alkalosis (rise in [HCO${}_{3}^{-}$]) (9). The type of acid-base disturbance encountered in OAs is a metabolic acidosis (Figure 1A). A wealth of information is now available on the effects of H^+^ ions on the heart (10–12). By acting as post-translational modifiers of proteins, H^+^ ions can alter biological functions by protonating amino acid residues, such as histidine. The low extracellular pH in uncompensated metabolic acidoses can modulate the heart by directly acting on surface-expressed proteins (13). Additionally, extracellular acidity can drive a fall in intracellular pH (14), and thus gain access to a myriad of intracellular targets, most of which show some degree of pH-sensitivity, including most elements of excitation-contraction coupling (10, 15, 16). For example, even minor reductions in pH_i_ from its physiological set-point (near 7.2) is capable of eliciting acute contractile depression, deranged Ca^2+^ handling, intracellular Na^+^ overload, and triggering of arrhythmias (10, 17).

In contrast, much less is known about the biological actions of the various organic anions associated with OAs. As organic acids accumulate in OAs, the concentrations of H^+^ ions and organic anions increase in tandem, but since the former is heavily buffered, its relative rise is orders of magnitude smaller. Whereas, the drop in pH during a metabolic acidosis may be equivalent to a sub-micromolar change in free [H^+^], the accumulation of organic anions in blood may reach millimolar levels (18–20) (Figure 1B). Furthermore, because the body's pH-regulatory apparatus is designed to sense and handle H^+^ ions, rather than organic anions, the latter may accumulate with little homeostatic oversight. OAs can result in the production of several different types of organic anions (e.g., lactate, propionate), and their collective concentration can be estimated from the so-called anion gap, usually calculated as [Na^+^] + [K^+^] – [Cl^−^] – [HCO${}_{3}^{-}$]. A high anion gap metabolic acidosis is a frequent observation in organic acidemias, attributable to a high collective concentration of organic acids that are not measured routinely in blood tests (9). A metabolic acidosis can become compensated by hyperventilation to reduce pCO_2_. Whereas, this compensatory maneuver can restore normal pH, albeit at a reduced pCO_2_ and [HCO${}_{3}^{-}$], it does not *per se* remove the organic anion disturbance (Figure 1A).

It is becoming increasingly clear that organic anions are not biologically inert (21–24), and at least some of the common presentations of OAs are attributable to actions of these metabolites, rather than the pH *per se*. This notion is exemplified by the fact that not all OAs are associated with cardiac disease. For example, heart disease is not observed in isovaleric acidemia (25) or pyroglutamic acidemia (5-oxoprolinemia) (26), two examples of OAs associated with profound metabolic acidosis.

Studies of OAs provide a unique opportunity to appreciate the biological actions of organic anions on integrated biological systems. Here, we provide a historical account of all known OAs that feature cardiac complications, starting with the first documented case. We begin by presenting the commonest OAs and aim to present the remaining disorders grouped by the affected metabolic pathway. In order to better understand the pathophysiology of cardiac dysfunction in OAs, we take a focused approach on PA, the organic acidemia that is most strongly associated with heart disease, and which has been subject to a relatively larger number of mechanistic studies.

## Historical Overview of Organic Acidemias With Cardiac Complications

**Figure 2:**
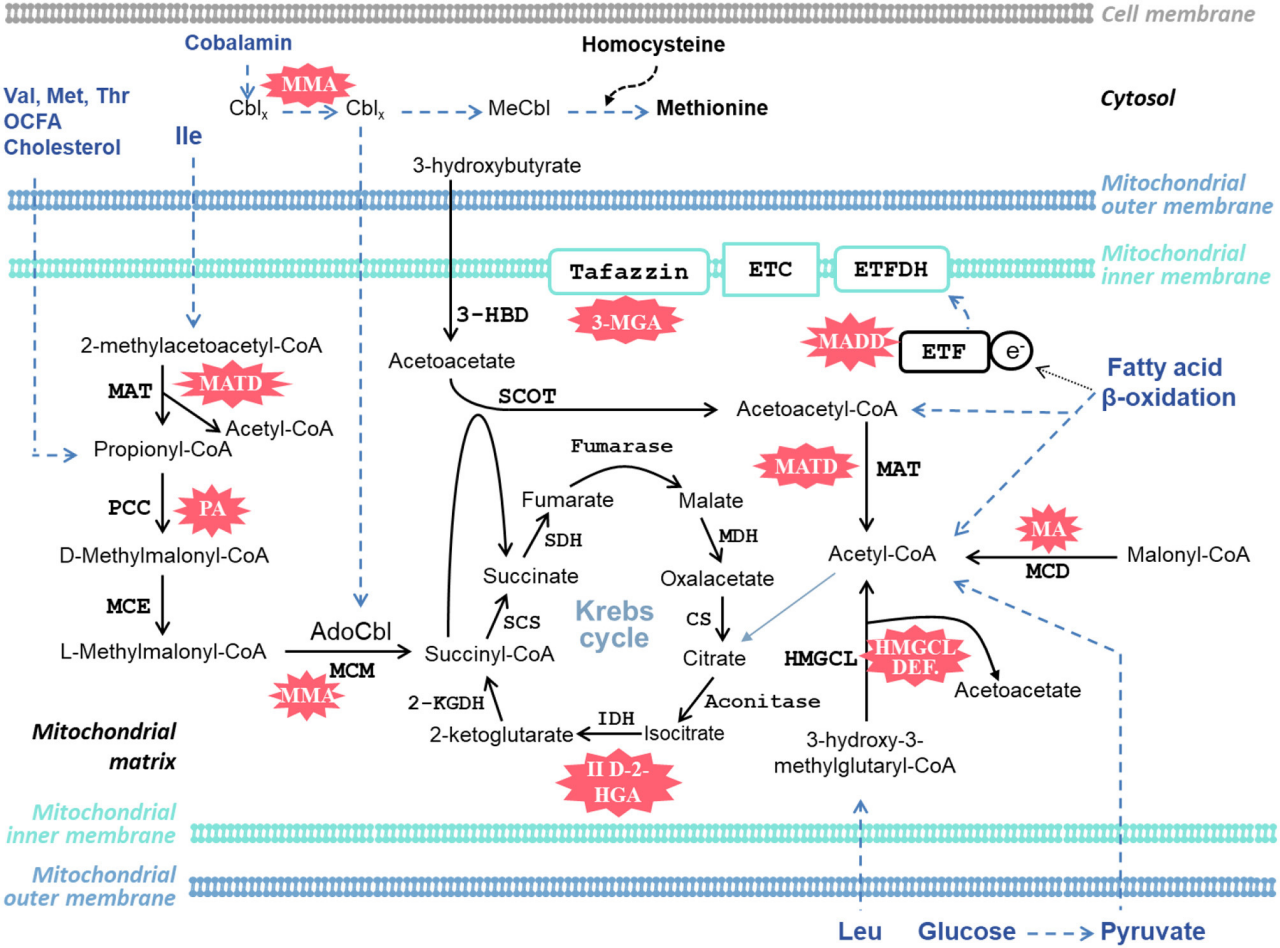
Schematic diagram of selected metabolic pathways in the heart that are affected and implicated in the organic acidemias discussed in this article. AdoCbl, adenosylcobalamin; Cbl, cobalamin; 2-KGDH, 2-ketoglutarate dehydrogenase; CS, citrate synthase; ETC, electron transport chain; ETF, electron-transfer flavoprotein; ETFDH, electron-transfer flavoprotein dehydrogenase; II D-2-HGA, type II D-2-hydroxyglutaric aciduria; 3-HBD, 3-hydroxybutyrate dehydrogenase; HMGCL, 3-hydroxy-3-methylglutaryl-CoA lyase; IDH, isocitrate dehydrogenase; Ile, isoleucine; Leu, Leucine; MA, malonic acidemia; MADD, multiple acyl-CoA dehydrogenase deficiency; MATD, mitochondrial acetoacetyl-CoA thiolase deficiency; MCD, malonyl-CoA decarboxylase; MCE, methylmalonyl-CoA epimerase (methylmalonyl-CoA racemase); MCM, methylmalonyl-CoA mutase; MDH, malate dehydrogenase; MeCbl, methylcobalamin; Met, methionine; 3-MGA, 3-methylglutaconic aciduria; MMA, methylmalonic acidemia; PA, propionic acidemia; PCC, propionyl-CoA carboxylase; SCOT, succinyl-CoA:3-ketoacid CoA transferase; SCS, succinyl-CoA synthetase (succinyl-CoA ligase); SDH, succinate dehydrogenase; Thr, threonine; Val, valine. Cbl_x_ refers to different oxidation states of the central cobalt ion in cobalamin. Blue dashed lines indicate several metabolic steps.

Cardiac complications have been reported in several organic acidemias. For completion, we briefly describe all OAs associated with cardiac disease, irrespective of whether they are a frequent complication or only reported sporadically (Table 1). The discovery of each OA is described, along with noteworthy case studies describing cardiac complications. The metabolic pathways implicated in the OAs discussed in this *Review* are illustrated schematically in Figure 2.

**Table 1:** Reference table of the main OAs known to associate with cardiac dysfunction.

**Disease name** *Other names*	**Incidence**	**Genes**	**OMIM**	**Cardiac sequelae observed**								
				**DCM**	**HCM**	**HF**	**LVH**	**LVNC**	**LQTS**	**VT**	**VF**	**TdP**
***SYSTEMIC OAs***												
**Propionic acidemia (PA)** - *Propionic aciduria*	1/2,000–150,000 live births	*PCCA* *PCCB*	606054	✓	✓	✓			✓		✓	
**Methylmalonic acidemia (MMA)** - *Isolated methylmalonic aciduria*	1/50,000–100,000 live births	*MMUT* *MMAA* *MMAB*	251000 251100 251110	✓	✓	✓					✓	
**3-methylglutaconic aciduria (3-MGA) type II** - *Barth syndrome*	~490 cases	*TAZ*	302060	✓	✓	✓	✓	✓		✓	✓	✓
**Multiple acyl-CoA dehydrogenase deficiency (MADD)** - *Glutaric aciduria type II*	1/200,000 live-births	*ETFA* *ETFB* *ETFDH*	231680	✓		✓	✓					
***CEREBRAL OAs***												
**Type II D-2-hydroxyglutaric aciduria (Type II D-2-HGA)**	~150 cases	*IDH2*	613657	✓	✓							
**Malonic acidemia (MA)** - *Malonic aciduria* - *Malonyl-CoA decarboxylase deficiency*	~30 cases	*MLYCD*	248360	✓	✓	✓	✓	✓				
***KETOLYTIC/KETOGENIC OAs***												
**3-hydroxy-3-methylglutaryl-CoA lyase (HMGCL) deficiency** - *HMG-CoA lyase deficiency* - *3-hydroxy-3-methyl glutaric aciduria*	~215 cases	*HMGCL*	246450	✓	✓	✓				✓	✓	
**Mitochondrial acetoacetyl-CoA thiolase (MAT) deficiency** - *β-ketothiolase deficiency* - *T2 deficiency*	~245 cases	*ACAT1*	203750	✓		✓						

*Whilst cardiac dysfunction has been reported in both of the commonest OAs, PA and MMA, cardiac disease only occurs commonly in PA. Collated information from the U.S. NIH National Library of Medicine Genetics Home Reference, Schwartz et al. (27), Chapman (3), and cited reports as per main text*.

*HF, heart failure; DCM, dilated cardiomyopathy; HCM, hypertrophic cardiomyopathy; LQTS, long QT syndrome; LVH, left-ventricular hypertrophy; LVNC, left-ventricular non-compaction cardiomyopathy; OA, organic acidemia/aciduria; OMIM, Online Mendelian Inheritance in Man; TdP, torsades de pointes; VT, ventricular tachycardia; VF, ventricular fibrillation*.

## Systemic Organic Acidemias

### Methylmalonic Acidemia (MMA) and Propionic Acidemia (PA)

#### Metabolic Lesion and Presentation

MMA and PA are amongst the most frequent OAs and are often considered in tandem because their enzymatic defects occur along the same metabolic pathway. MMA and PA are inborn errors of the catabolism of propionate, a short-chain fatty acid, from branched chain amino acids (valine, isoleucine, methionine, threonine), odd-chain fatty acids (OCFAs), and cholesterol (28). PA is caused by defects in propionyl-CoA carboxylase (PCC), a biotin-dependent enzyme that carboxylates propionyl-CoA to D-methylmalonyl-CoA. PCC is a α6β6 heterododecamer composed of PCCA and PCCB subunits, encoded by *PCCA* and *PCCB*, respectively, and PA can result from mutations in either gene (29). D-methylmalonyl-CoA is subsequently racemized by methylmalonyl-CoA epimerase (methylmalonyl-CoA racemase) to L-methylmalonyl-CoA. Disorders of MMA includes a heterogeneous group of disorders caused by several different deficiencies: (1) in methylmalonyl-CoA epimerase; (2) in L-methylmalonyl-CoA mutase (MCM), which isomerizes L-methylmalonyl-CoA to succinyl-CoA; (3) defects in cobalamin absorption or intracellular trafficking; (4) intracellular defects involving the distal steps of the cobalamin processing pathway (cblA, cblB, cblD2) impacting solely on the synthesis of the co-factor adenosylcobalamin essential for MCM activity, resulting in isolated MMA; (5) intracellular defects involving the proximal steps in the cobalamin processing pathway (cblC, cblD, cblE, cblF, cblG, cblJ) impacting on the synthesis of both methylcobalamin, the co-factor for methionine synthase, and adenosylcobalamin, the co-factor for MCM activity, resulting in combined MMA and homocystinuria (28, 30).

The reduction in MCM activity causes the characteristic systemic accumulation of methylmalonyl-CoA and methylmalonic acid. However, because of the proximity of the biochemical lesion to PA, MMA shares many of the metabolites that are detected during plasma and urinary screening, including propionyl-carnitine, 2-methylcitrate, 3-hydroxypropionate, propionyl-glycine, and glycine (5). MMA and PA generally manifest with a more severe early-onset neonatal form, and a milder late-onset form presenting later in infancy, childhood, or adolescence (although there is no clear definition of what is considered “late”) (5). Their initial clinical presentations are similar. In the neonatal onset form, patients typically present with pancytopenia and encephalopathy, as well as a string of metabolic disturbances, including high anion gap metabolic acidosis, ketoacidosis, and hyperammonemia (5). Chronically, MMA and PA typically present with similar signs and symptoms, such as developmental delay, dystonia, recurrent vomiting with ketoacidosis, and failure to thrive. However, they tend to differ clinically, in that MMA patients predominantly develop chronic renal failure, whereas PA patients commonly present with cardiomyopathy and prolonged QT intervals (5, 28).

Cardiac disease has been noted in MMA. Prada et al. (31) reported HCM in a 22-year-old who died of ventricular fibrillation (VF)-induced cardiac arrest (see Box 1 and Box 2). In the same series, they documented a 4-years-old who had paroxysmal supraventricular tachycardia and heart failure (HF) secondary to DCM, who died due to cardiorespiratory arrest. It should be noted however that cardiac disease is not common in MMA. In a large case study of 273 MMA patients, only 4 (~1%) had cardiomyopathy (32). In contrast, cardiomyopathy is a lot more common in PA, with an incidence rate of ~9–23% (28), whilst the incidence of long QT syndrome has been reported to be as high as 70% (33). Since PA is a focus of this *Review*, we have dedicated an entire section *(Cardiac Dysfunction In Propionic Acidemia)* on providing a detailed account and analysis of the cardiac complications encountered in PA.

Box 1 **Glossary of cardiomyopathies encountered in OAs.**

Around 5% of all IMDs are associated with cardiomyopathy (156), a group of cardiac muscle disorders characterized by abnormal chamber size, wall thickness and/or contractile function (157). Classifications of cardiomyopathies are known by either AHA/ACC (American Heart Association/American College of Cardiology) or ESC (European Society of Cardiology) standards (142, 158). The types of cardiomyopathy associated closely with OAs include dilated (DCM), hypokinetic non-dilated (HNDC), hypertrophic (HCM), and left-ventricular non-compaction (LVNC) cardiomyopathies (142).

HCM is defined as the presence of a hypertrophied, non-dilated ventricle, in the absence of hemodynamic causes that are capable in eliciting the wall thickening (e.g., physiological hypertrophy due to exercise, or pathological hypertrophy due to aortic stenosis) (158). Common histological findings include myocyte disarray and fibrosis (152). The genetic causes of HCM are almost exclusively due to mutations of sarcomeric genes, unlike DCM. Although HCM has been associated with several IMDs (158), it is not frequently observed in OAs.

HNDC is a relatively new category introduced to describe patients that have LV or biventricular systolic dysfunction (hypokinesia, or decreased “wall-motion”), in the absence of dilatation or hypertrophy, or abnormal loading conditions and coronary artery disease (142).

DCM is defined by the presence of left-ventricular (LV) or biventricular dilatation, and systolic dysfunction (based on ejection fraction; EF), in the absence of abnormal loading conditions (hypertension, valve disease), or coronary artery disease. DCM is a common type of cardiomyopathy worldwide and is the most frequent form observed in IMDs. DCM can occur due to disorders isolated to the heart, for example from mutations in classical “disease genes” such as the sarcomeric proteins titin (*TTN*) and myosin heavy chain 7 (*MYH7*). However, unlike HCM, it can also be due to mutations in genes accounting for different aspects of cell function, such as those involved in energy production, myofilament activation and Ca^2+^ handling (152). Notably, DCM can also occur due myocardial damage secondary to systemic diseases, such as metabolic disorders (142, 143, 158). Key complications with DCM include arrhythmias, decompensated heart failure (HF), and cardiogenic shock (159).

LVNC is characterized by an outer compacted myocardial layer, and an inner non-compacted layer featuring prominent trabeculations coincident with large intertrabecular recesses (160). The pathophysiology of LVNC is fascinating in its own right. Trabeculae are finger-like projections of the myocardium and are a distinguishing feature of the developing embryonic heart. Since the early developing heart is devoid of coronary vascularization, the diffusion of nutrients and oxygen to the embryonic myocardium is achieved by increasing surface area through the formation of trabeculae. During the later stages of chamber maturation, the trabeculae itself undergoes remodeling (compaction) whereby they collapse, compact, and the spaces between the trabeculae gives rise to capillaries (161). However, in LVNC, this compaction phase fails, leading to the characteristic trabeculae that can be seen on cardiac imaging modalities.

Box 2 **Glossary of arrhythmias encountered in OAs.**

Cardiac arrhythmias can be grossly classified as supraventricular or ventricular based on their anatomical site of origin. Ventricular arrhythmias can be life-threatening as they can rapidly evolve to result in hemodynamic instability and if untreated, cardiac arrest and sudden cardiac death (SCD). The ventricular arrhythmias frequently observed in OAs are ventricular tachycardia (VT) and ventricular fibrillation (VF). Though technically VF is the “lethal” ventricular arrhythmia, VT can also be dangerous, as in the absence of prompt treatment, it can progress to VF and circulatory arrest. Ventricular arrhythmias usually occur due to abnormal structural substrates (e.g. infarction, cardiomyopathy) or electrophysiological substrates (e.g. long QT) in the myocardium.

Long QT syndrome (LQTS) is characterized by prolongation of the QT interval in the electrocardiogram (EKG), and occurs due to abnormal cardiac repolarization. The QT interval is related to the action potential duration. It is normally reported as corrected QT (QTc), which normalizes for variation in heart rate, since action potential duration changes with pacing frequency. QT prolongation is a major pro-arrhythmic substrate that can cause sudden cardiac death from life-threatening arrhythmias, notably VF and torsades de pointes (TdP; polymorphic ventricular tachycardia) (162).

LQTS can either be congenital or acquired. There are 15 congenital LQT sub-types (LQT1-LQT15), each associated with their own gene. The majority of congenital LQTS is accounted for by mutations in *KCNQ1* (*I*_Ks_), *KCNH2* (*I*_Kr_), or *SCN5A* (*I*_Na_) (163). Acquired LQTS, the most likely form to occur in OAs, can be due to a number of causes, including bradycardia, drugs, and electrolyte disturbances (150). It is also known that additional non-genetic factors (demographic and environmental) can act as disease modifiers in LQTS, including for example, autonomic nervous system neurotransmitter signaling (149). It has been shown that the KCNH2 channel (hERG; *I*_Kr_) accounts for almost all cases of acquired LQTS (162, 164).

The seminal report describing MMA was made by Oberholzer et al. (34) in two unrelated infants, and included the results of a thorough metabolic workup enabling identification of the metabolic defect. The discovery of PA was the least straightforward of all the OAs, spanning a decade of research between the 60 and 70's. The index patient was a Caucasian male born in 1957 and admitted to Johns Hopkins Hospital at 8 months of age with numerous signs and symptoms, including vomiting, intellectual disability, ketosis, hyperglycinemia and hyperglycinuria in the neonatal period. The seminal report was published by Dr. Barton Childs in 1961, with the disorder initially being referred to as “idiopathic hyperglycinemia,” based on the most unusual biochemical finding being significantly raised plasma glycine (35). Their key observation was that when specific amino acids were administered (leucine, isoleucine, valine, threonine, methionine), the patient's symptoms worsened, whereas a decrease in blood acidosis, glycine, and ketones was observed when these amino acids were restricted in the diet (36).

Interestingly, the first hint of the basic defect underlying idiopathic hyperglycinemia was borne not from studying the disease itself, but rather the closely related, MMA. Following the discovery of MMA by Oberholzer et al. (34), a study published by a team from Yale (37) provided speculation that the primary biochemical lesion in idiopathic hyperglycinemia was at the level of PCC.

The first report actually using a term close to PA was a case report published by Hommes et al. (38) from the Netherlands, describing fatal “propionic acidemia” in a male neonate with severe metabolic acidosis and very high propionic acid in blood (5.4 mmol/L). The boy presented at 60 h of age with hyperventilation and marked hypotonia but died at 104 h of age. Initial laboratory studies showed a mild ketosis, and notably a severe metabolic acidosis (pH 6.98). Importantly, Hommes et al. applied the gas-liquid chromatography (GLC) method developed by Tanaka et al. (39) employed in the discovery of isovaleric acidemia (another OA), to measure plasma short-chain fatty acids. Although isovaleric acid was absent, there was a large peak coinciding with that of propionic acid, which was calculated as being 40,000 μg/100 mL (5.4 mmol/L) using the chromatogram peak area; Tanaka et al. (39) reported a normal value of 28 μg/100 mL (3.8 μmol/L) propionic acid in healthy control children. Based on this observation, and given that a biosynthetic route of propionate is through the oxidation of OCFAs, Hommes et al. performed GLC for free fatty acids and triglycerides on post-mortem liver tissue, finding that C_15_/C_17_ OCFAs were raised. Taken together, given the block in conversion of propionic acid to methylmalonic acid, and the increased number of OCFAs (C_15_/C_17_) detected in liver, the authors postulated that the observed disorder was consequent to an absence of PCC.

Subsequent reports consolidated on the findings by Hommes et al. (38), although their coining of “propionic acidemia” was not immediately adopted by all research groups, with some authors still referring to it as idiopathic hyperglycinemia. Hsia et al. (40) published a study on propionate metabolism in peripheral leukocytes obtained from a patient with “ketotic hyperglycinemia,” and one with MMA. The authors had renamed the term idiopathic hyperglycinemia to distinguish the condition from other diseases that manifest hyperglycinemia, but without ketoacidosis (41). In leukocytes from a ketotic hyperglycinemia patient, Hsia et al. (40) found normal metabolism of methylmalonate, but a failure to metabolize propionate. In contrast, leukocytes from an MMA patient could not metabolize methylmalonate, but propionate could be successfully metabolized. The authors concluded that the biochemical lesion in ketotic hyperglycinemia (i.e., PA) was in the carboxylation of methylmalonate and speculated this was due to defective PCC.

The study by Gompertz et al. (42) performed PCC and MCM enzyme activity assays on mitochondria isolated from post-mortem liver in a patient with fatal “propionic acidemia.” Gompertz et al. found the level of PCC in the patient to be 9.7% that of controls, whilst MCM activity was normal, definitively localizing the enzymatic defect in PA to PCC. In a subsequent study, Hsia et al. (43) performed flux isotope tracer studies in cultured fibroblasts from a patient with “ketotic hyperglycinemia” and found that they were unable to oxidize propionate-^14^C to ^14^CO_2_, but oxidized methylmalonate-^14^C and succinate-^14^C normally. PCC activity was determined to be <2% normal activity in the fibroblasts. Furthermore, PCC activity was ~50% of normal in both parents of the patient. Based on the collective evidence from their study, combined with the work of Gompertz et al. (42), Hsia et al. (43) concluded that “ketotic hyperglycinemia” and “propionic acidemia” are the same disease, caused by defective PCC, and that the defect is inherited as an autosomal recessive trait.

Although propionate and propionyl-CoA are the first and primary metabolites to accumulate with PCC dysfunction, they are challenging substances to measure accurately from a technical standpoint. Thus, the diagnosis of PA has relied on more downstream metabolites directly borne from excess propionate. The markers used clinically today to diagnose PA include elevations of propionyl-carnitine, 2-methylcitrate, 3-hydroxypropionate and propionyl-glycine in physiological fluids (28, 44). Isotope tracer studies are frequently employed to study propionate metabolism (or rather, IMDs in general), and the discovery of these markers were borne from such studies. The use of 3-hydroxypropionate and 2-methylcitrate as clinical markers of PA were initially reported from the laboratory of Dr. William Nyhan in the 1970's through studying the metabolic flux of [*1*-^14^C] propionate intravenously infused into PA and MMA patients (45, 46). Indeed, these markers have stood the test of time, being the principle biochemical markers by which PA is diagnosed today.

### 3-Methylglutaconic Aciduria Type II (Barth Syndrome)

#### Metabolic Lesion and Presentation

3-methylglutaconic aciduria (3-MGA) type II, more commonly referred to as Barth syndrome (BTHS), is a systemic OA caused by mutations in the *TAZ* gene that codes for tafazzin (previously known as G4.5). It is X-linked, occurring almost exclusively in males (although one female has been reported who had the mutation in both X chromosomes) (47). BTHS results from a loss-of-function mutation of tafazzin, an acyl-transferase located in the inner mitochondrial membrane that catalyzes the remodeling of cardiolipin; cardiolipin has been implicated in numerous aspects of physiology, but is critically important in maintaining mitochondrial structure and apoptosis (47, 48). Patients with BTHS have increased urinary excretion of 3-methylglutaconic acid, and typically present with a triad of key clinical features: dilated cardiomyopathy (DCM), neutropenia and skeletal myopathy (47). Additionally, BTHS has been associated with numerous cardiac pathologies, including left ventricular non-compaction cardiomyopathy (LVNC) and ventricular arrhythmias, as discussed further below.

In the first study of its kind, examination of the BTHS registry in 2012 found that ~70% of BTHS patients have a form of cardiomyopathy in their 1st year of life (49). We highlight below a few reports only, including the historical study by Dr. Barth, and reports describing common cardiac complications seen in BTHS (DCM, LVNC, ventricular arrhythmias). We refer the reader to reviews by Clarke et al. (47) and Jefferies (48) for more comprehensive overviews on cardiac complications encountered in BTHS.

BTHS was first described in a preliminary communication by Dr. Peter Barth in 1981 and in the subsequent publication (50), which reported the death of three boys from a large Dutch family in the neonatal or infancy period due to cardiac failure or septicemia, despite normal delivery and full-term gestation. One boy (“case V-11”) presented at 4 days of age with severe metabolic acidosis (arterial pH 6.94), cardiomegaly (chest X-ray) and high white cell counts. The patient died 3 h after admission due to sepsis, as confirmed by bacterial cultures from multiple sample types. Another boy (“case V-12”) had neutropenia on birth so was kept in hospital. Electrocardiogram (EKG) and chest X-ray were normal, but a progressive lactic acidosis was noted up till 10 days of age. After being discharged at 6 weeks of age, the patient presented at 15 months with dyspnea, hypothermia, hypoglycemia and coma. On examination his EKG was normal but had a gallop rhythm and significant cardiomegaly (presumably DCM), and died of cardiac arrest the same day.

#### Associated Cardiac Defects

DCM and chronic heart failure (CHF) are common findings in BTHS. One of the earlier published case series for these conditions were by Kelley et al. (51), in which the boys in this 7-patient series died either from HF or sepsis. “Patient 1” and “Patient 2” had signs of DCM and CHF at 2 days of age which was treated with positive inotropes (digoxin) and diuretics (furosemide). The boys had increased urinary 3-methylglutaconate and 3-methylglutarate and experienced very poor growth during their 1st year. Although their cardiac condition remained relatively stable, there was persistent DCM (biventricular enlargement) and low left ventricular (LV) fractional shortening (measure of contractility). “Patient 5” presented with respiratory distress at 3 months of age. Further investigation showed marked cardiomegaly, biventricular dilatation, and severely reduced LV fractional shortening. This patient was treated with carnitine, as well as digoxin and furosemide, but no improvement in cardiac size and function was seen at 4 years of age. “Patient 3” and “Patient 7” experienced a similar syndrome, albeit over different timelines.

LVNC is another common cardiomyopathy encountered in BTHS as described by Roberts et al. (49). Bleyl et al. (52) described a family in which 6 individuals exhibited cardiomegaly and HF. Aside from the sixth patient for which there is no data available, all other patients in this series had chamber dilatation and coarse trabeculation, indicative of LVNC.

A large observational cross-sectional study of cardiac complications in BTHS patients demonstrated that DCM, LVNC and ventricular arrhythmias remained a common finding in a more reflective pool of patients (53). Ventricular arrhythmias are particularly common in adolescents and young adults with BTHS. Spencer et al. (54) reported a series of 5 adolescent/young-adult BTHS patients with DCM and lethal ventricular arrhythmias. One boy, of 11 years with DCM, collapsed during play with cardiac arrest. Interestingly, torsades de pointes (polymorphic ventricular tachycardia) was documented for this patient. From his EKG, the corrected QT interval (QTc) was 444 ms, which could be considered as “borderline” elongated (55). He was managed with an implantable cardioverter defibrillator (ICD), and episodes of short-lived ventricular tachycardia (VT) and VF were noted.

### Multiple Acyl-CoA Dehydrogenase Deficiency (MADD; Glutaric Aciduria Type II)

#### Metabolic Lesion and Presentation

Multiple acyl-CoA dehydrogenase deficiency (MADD; also known as glutaric aciduria type II) is an inborn error of fatty acid oxidation and amino acid (lysine and tryptophan) catabolism. MADD can be caused by mutations in three genes: *ETFA* and *ETFB* (encoding for electron-transfer flavoprotein, ETF) and *ETFDH* (encoding for electron-transfer flavoprotein dehydrogenase, ETFDH) (56). The clinical phenotype can vary, but MADD patients can be broadly classified intro three groups according to disease severity: (1) neonatal onset with congenital anomalies; (2) neonatal onset without congenital anomalies; (3) mild and/or later onset (57). Regardless of disease severity, there will be some degree of glutaric acid accumulation in fluid compartments, as well as increased concentrations of medium- and long-chain acyl-carnitines. The neonatal onset form is typically more severe, presenting with metabolic acidosis, non-ketotic hypoglycemia, hyperammonemia and cardiomyopathy (56).

Przyrembel et al. (58) were the first to describe MADD (as glutaric aciduria type II) in a 2-hour-old neonate admitted to hospital due to tachypnea of 80 breaths per minute. At 16 h of age, the patient was in compensated metabolic acidosis with significant hypoglycemia. Despite glucose and sodium bicarbonate infusion, the patient had cardiorespiratory arrest at 32 h of age. Although resuscitation was successful, the metabolic acidosis and hypoglycemia persisted, leading to bradycardia and hypothermia. At 70 h, severe metabolic acidosis became apparent (blood pH 6.6), followed by cardiac arrest. Subsequent organic acid profiling found abnormally large elevations of glutaric acid in plasma and urine.

#### Associated Cardiac Defects

There are several reports describing cardiomyopathy in MADD (59–61). The study by van Hove et al. (59) described three patients with MADD, two of whom had cardiomyopathy, who were phenotypically rescued with D,L-3-hydroxybutyrate treatment. The authors postulated that the improvement was attributable to increased energy bioavailability in the heart in the form of ketone bodies (a source of energy that can be used by the heart without need for fatty acid oxidation, which is normally the energy source used by the heart).

## Cerebral Organic Acidemias

### Type II D-2-Hydroxyglutaric Aciduria (Type II D-2-HGA)

#### Metabolic Lesion and Presentation

2-hydroxyglutaric acid (2-HG) a five-carbon dicarboxylic acid, and due to the chirality of the hydroxyl group on the second carbon, it exists as enantiomeric dextro- (D) and levo (L) forms. 2-hydroxyglutaric aciduria (2-HGA) is unique, in that different enzymatic defects can result in the systemic accumulation of D-2-HG, L-2-HG, or a combination of these, to result in three distinct organic acidurias: L-2-hydroxyglutaric aciduria (L-2-HGA, caused by defects in the housekeeping enzyme L-2-hydroxyglutaric dehydrogenase, *L2HGDH*); Type I and Type II D-2-hydroxyglutaric aciduria (Type I and Type II D-2-HGA, see below for further details); combined D,L-2-hydroxyglutaric aciduria (D,L-2-HGA). GC-MS or LC-MS/MS is required for chiral differentiation during diagnosis (62).

It is remarkable that the simple chirality of 2-HG results in some shared features, but also markedly distinct clinical presentations. Both D-2-HGA and L-2-HGA patients typically present with unexplained developmental delay and/or neurological dysfunction (62). However, whilst L-2-HGA is generally associated with neurological signs (e.g., intellectual disability, motor developmental delay, extrapyramidal symptoms), D-2-HGA, and specifically Type II D-2-HGA, tends to present earlier in life with more severe symptoms (vs. its Type I counterpart), and commonly manifests with DCM. Although both types of D-2-HGA share the accumulation of D-2-HG in physiological fluids, they have genetically distinct defects. Type I D-2-HGA is caused by mutations in *D2HGDH*, causing defects in D-2-hydroxyglutarate dehydrogenase (63), whereas Type II D-2-HGA, is caused by gain-of-function mutations in *IDH2* (encoding for isocitrate dehydrogenase 2) (64).

The first report of L-2-HGA was by Duran et al. (65). D-2-HGA was described in the same year by Chalmers et al. (66). Combined D,L-2-HGA was described several years later by Muntau et al. (67). Combined D,L-2-HGA is caused by impaired function of the mitochondrial citrate carrier (CIC) due to pathogenic mutations in *SLC25A1* (68), however cardiac disease is not known to occur in D,L-2-HGA (69). D-2-HGA was not recognized as a distinct neurometabolic disorder until the late 90's. In the study by van der Knaap et al. (70), eight patients were identified with D-2-HGA. In a recent article by Kranendijk et al. (62), the authors included their own observations on 14 Type I and 19 Type II D-2-HGA patients. In this cohort, no Type I patient had cardiomyopathy. Of the 19 Type II patients, nine had cardiomyopathy, with most of these being dilated.

### Malonic Acidemia (MA)

#### Metabolic Lesion and Presentation

Malonic acidemia (MA) is a cerebral OA occurring as a result of malonyl-CoA decarboxylase (MCD) deficiency and is caused by pathogenic variants in *MLYCD* (71). The disease phenotype is variable, but patients typically present with developmental delay, seizures, as well as cardiomyopathy. Laboratory findings can include metabolic acidosis and hypoglycemia. Typical biochemical abnormalities include significant accumulation of malonyl-carnitine and malonic acid in physiological fluids, and sometimes, combined elevations of malonic and methylmalonic acids (72). Malonyl-CoA is unique in that it is an important and dynamic regulator of fatty acid oxidation (FAO). At high concentrations, it inhibits carnitine palmitoyl-transferase 1 (CPT1), an enzyme that mediates mitochondrial FAO by converting acyl-CoAs into acyl-carnitines. Conversely, a fall in malonyl-CoA activates CPT1, FAO, and specifically in hepatic tissue, ketogenesis (73). It is especially important to maintain a normal level of malonyl-CoA in the heart since it is an organ primarily reliant on FAO as its primary fuel source (74); although in certain specific pathological circumstances such as ischemia-reperfusion injury, inhibiting FAO via malonyl-CoA is regarded as being cardioprotective (75). In MA, there is some evidence to suggest that cardiomyopathy can be improved by supplementing the diet with medium-chain triglycerides, which can be converted into ketone bodies by the liver and utilized by the heart as an energy source (76).

#### Associated Cardiac Defects

Between 1984, when MA was first reported, and 2014, fewer than 30 cases have been described (77). The first case of MA was described by Brown et al. (78) in a 5-year-old boy presenting with short-stature, abdominal pain and vomiting. The first case of cardiomyopathy was reported in a short communication by Matalon et al. (79) in a 7-month-old boy who presented with mild developmental delay and hypotonia. The infant had excessive levels of urinary malonic, methylmalonic, and ethylmalonic acids, accompanied by an abnormally high ratio of esterified-to-free carnitine (>15-fold), with malonyl-CoA decarboxylase determined as being ~30% of normal wild-type activity. M-mode echocardiography revealed LV dilatation (increased LV diastolic dimension) and reduced contractile function, indicative of DCM.

Cardiac involvement is common in MA. In a case series of 8 new patients, heart disease (including cardiomyopathy, CHF, mild LV hypertrophy) was reported in three patients, equivalent to an incidence rate of 38% (71). Salomons et al. additionally reviewed all 15 previously reported cases, which also had similar incidence of cardiomyopathy (40%; 6/15). It is worth noting that CHF due to HCM has also been reported in MA, in a 2-month-old boy presenting with severe tachypnea (80). Left chest lead T-wave inversion and borderline QT interval prolongation was also reported for this infant. Finally, more recently, mild LVNC has been noted in a 1-year-old boy identified by newborn screening, although his cardiac function was normal (81).

## Ketolytic/Ketogenic Organic Acidemias

### 3-Hydroxy-3-Methylglutaryl-CoA Lyase (HMGCL) Deficiency

#### Metabolic Lesion and Presentation

3-hydroxy-3-methylglutaryl-CoA lyase (HMGCL) deficiency or 3-hydroxy-3-methylglutaric aciduria results from mutations in the *HMGCL* genes, and is an OA affecting leucine metabolism. HMGCL is also required for the synthesis of the ketone bodies acetoacetate and 3-hydroxy-*n*-butyrate (82). HMGCL catalyzes the last step in leucine catabolism involving the cleavage of 3-hydroxy-3-methylglutaryl CoA to acetoacetate and acetyl-CoA (83). Patients typically present in the neonatal period with acute metabolic decompensation and non-specific symptoms such as vomiting and seizures. Key laboratory findings include metabolic acidosis, raised anion gap, hypoglycemia, abnormal blood acyl-carnitines, as well as 3-hydroxy-3-methylglutaric acid and its derivatives in urine (84).

HMGCL deficiency was first described by Faull et al. (85) in a 7 month-old neonate presenting with diarrhea, vomiting, drowsiness and apnea. In this patient, initial laboratory investigations showed metabolic acidosis and hypoglycemia. Out of a total 211 cases of HMGCL deficiency identified and reviewed in a recent meta-analysis by Grünert and Sass (82), cardiac complications were reported in four cases. Grünert et al. (84) reported DCM and cardiac arrest in one patient from a larger case series of 37 patients.

#### Associated Cardiac Defects

The first case was fatal arrhythmia associated with cardiomyopathy in a 7-month-old boy reported by Gibson et al. (86). The child originally presented at 4 months of age with vomiting, respiratory arrest, hypoglycemia, hyperammonemia and metabolic acidosis. No cardiac abnormalities were present, but at 7 months of age, he was re-hospitalized due to metabolic decompensation and febrile upper respiratory illness. On examination he had VT that rapidly progressed to VF, coincident with poor LV function on echocardiography and cardiomegaly on chest X-ray. The patient died 50 h after admission despite aggressive resuscitation. There was no evidence of myocarditis, but four-chamber dilatation and mild pulmonary edema was noted on autopsy, indicative of DCM.

Leung et al. (87) reported a 23-year-old adult male with HMGCL deficiency and acute HF secondary to DCM. The patient was diagnosed with HMGCL deficiency at 9 months of age and was managed conservatively with L-carnitine, and was largely absent of symptoms. He presented at the age of 23 following a 2-month history of exertional dyspnea and progressive malaise. Urinary organic acid analysis showed significant amounts of 3-hydroxy-3-methylglutaric and methylglutaconic acids. Brain natriuretic peptide (BNP) was elevated indicative of myocardial strain, although cardiac troponin I was undetectable, thus excluding overt myocardial injury (88). EKG showed sinus tachycardia and chest X-ray showed cardiomegaly. Further investigation using CMR demonstrated poor ejection fraction (EF; 15%), severe left-ventricular (LV) enlargement (significantly raised LVEDV), severe right-ventricular dysfunction and bi-atrial enlargement. The patient was diagnosed with HF secondary to DCM.

Most often, IEMs and OAs are associated with cardiomyopathy, which is typically dilated. However, Köksal et al. (89) interestingly reported LVNC in an 8 month-old boy with HMGCL deficiency. The patient presented with vomiting, respiratory distress and tonic-clonic seizures. Laboratory investigations showed metabolic acidosis, hypoglycemia, hyperammonemia, and raised urinary organic acids characteristic of HMGCL deficiency. LVNC was revealed upon echocardiography.

### 2-methylacetoacetyl-CoA Thiolase (MAT) Deficiency (β-Ketothiolase)

#### Metabolic Lesion and Presentation

2-methylacetoacetyl-CoA thiolase (MAT; also known as β-ketothiolase, acetoacetyl-CoA thiolase, T2) deficiency results from pathogenic variants in *ACAT1*, and is an organic aciduria involving defects in ketone body metabolism (ketolysis) and BCAA catabolism (90). MAT is involved in two metabolic pathways: (1) isoleucine catabolism, where it cleaves 2-methylacetoacetyl-CoA to propionyl-CoA and acetyl-CoA; (2) utilization of ketone bodies (ketolysis), cleaving acetoacetyl-CoA to acetyl-CoA (91). MAT deficiency was first described by Daum et al. (92) as β-ketothiolase deficiency in a 6-year-old boy with severe metabolic acidosis and significant concentrations of the organic acids α-methyl-β-hydroxybutyric acid and α-methylacetoacetate in urine. Acetoacetate, which is normally raised in MAT deficiency, was not commented upon in this article.

Most patients typically present with a ketoacidotic crisis between 6 and 36 months of age but are largely asymptomatic between intermittent ketoacidoses. It can present clinically similar to ketotic hypoglycaemia and succinyl-CoA:3-ketoacid CoA transferase (SCOT) deficiency (see next *Section*), thus enzymatic diagnosis to confirm MAT deficiency is important (90). In total, 244 patients have been identified and reported thus far (91) but only one case had cardiac complications, an 8-year-old girl who died due to HF secondary to severe DCM (93).

### Other Organic Acidemias With Reported Cardiac Involvement

Other organic acidemias/acidurias that have reported cardiac complications in a handful of patients, with little information, are briefly mentioned in this section for completion.

Mevalonic aciduria is considered a cerebral organic acidemia (3). It results from a deficiency in mevalonate kinase (involved in the early steps of cholesterol biosynthesis), however a defect in mevalonate kinase can also result in hyperimmunoglobulinemia D syndrome. The typical biochemical finding is elevated mevalonic acid and mevalonolactone (94). It was first described by Hoffmann et al. (95) in a 2-year-old boy presenting with severe failure-to-thrive, developmental delay and dysmorphic features, with massive increases in both urinary and plasma mevalonic acid. In a case series of 11 patients by Hoffmann et al. (96), cardiomyopathy and heart block was identified in one of these patients.

Succinyl-CoA:3-ketoacid CoA transferase (SCOT) catalyzes the first, and rate-limiting step of ketone body utilization (ketolysis) by activating acetoacetate to acetoacetyl-CoA; acetoacetyl-CoA is then metabolized by acetoacetyl-CoA thiolase into two acetyl-CoA molecules to enter the Krebs cycle (97). SCOT deficiency is caused by pathogenic variants in *OXCT1*, resulting in the inability of extra-hepatic organs to utilize ketone bodies, and thus patients experience episodic ketoacidosis (98). In a case series by Saudubray et al. (99), the authors claimed that one patient (6 month-old boy) with SCOT deficiency had cardiomegaly, although no other information was reported. It is unlikely cardiac disease is a consistent observation in this disease.

Although not strictly an OA, succinyl-CoA ligase (SUCL) deficiency is an IMD caused by mutations in *SUCLA2* and *SUCLG1*, and is associated with a methylmalonic aciduria (100). SUCL catalyzes the conversion of succinyl-CoA and ADP or GDP, to succinate and ATP or GTP. SUCLG1 is ubiquitously expressed with the highest expression in heart, brain, kidney and liver, and 14% of patients with *SUCLG1* mutations from a large case series of 71 patients presented with HCM (100).

## Cardiac Dysfunction in Propionic Acidemia

Among the OAs, PA is most commonly associated with cardiac disease. Further, it is one of the most frequent OAs, and therefore has received a greater share of research interest from the scientific community. A detailed description of cardiac dysfunction in PA is therefore provided in this *Section*.

### First Observations and Typical Clinical Features of Heart Disease in PA

Heart disease in PA was first reported by Massoud and Leonard (101) in a series of 19 pediatric patients. Six individuals had evidence for cardiomyopathy, one of whom (Case 6) also had a prolonged QT. This patient presented in the neonatal period with metabolic acidosis, emesis and hypotonia. She was diagnosed with PA at 3 days of age and managed conservatively. At 4 years of age, the patient presented with a 2-months history of breathlessness, skeletal myopathy and cardiomyopathy. EKG findings included ST segment depression and prolonged QT intervals (although duration was not reported). On echocardiography, reduced contractile function was noted, and after developing acute HF, the patient died.

As eluded to, cardiac complications account for significant morbidity and mortality in PA patients, most commonly in the form of cardiomyopathy and prolonged QT intervals (28, 102). Although HCM (103) and possibly LVNC (104) have been reported, patients typically have DCM. In one of the largest case series to date, cardiomyopathy had a prevalence rate of 23% occurring in six out of 26 patients, all of whom had DCM, with a mean age of the onset of 7 years (105).

More recently, a longitudinal observational monocentric study of 18 PA patients spanning 28 years was published by Kovacevic et al. (106). Here, patients were categorized into “early” or “late-onset” (PA symptoms within 28 days, or after 28 days of life). Most patients (14/17; 82%) had an early-onset form of PA, and cardiomyopathy was seen in 7 patients (7/18; 39%), all of whom were from the early-onset cohort. Two of the patients with cardiomyopathy (2/18; 11%) had DCM (LV dysfunction and dilatation) whilst 5 patients (5/18; 28%) had hypokinetic non-dilated cardiomyopathy (HNDC; LV dysfunction only). QTc in these patients were on average normal (445 ± 18 ms). Interestingly, there was no difference in QTc between early-onset and late-onset patients, and no ventricular arrhythmias were recorded in either group. Furthermore, longer QTc was associated with a larger LV end-diastolic diameter (LVEDD), and critically, the number of metabolic decompensations per case had no significant effect on either the onset of cardiomyopathy or QTc duration. The latter point supports the idea that PA-induced cardiac dysfunction is uniquely due to propionate and its metabolites, as opposed to an effect due to pH disturbances.

Interestingly, diastolic function was also assessed in the study by Kovacevic et al. (106) using Doppler echocardiography. To the best of our knowledge, this is the first clinical study in PA patients to evaluate diastolic function. Pulsed-wave Doppler was used to measure the mitral E/A wave ratio, a marker determined by the left atrial (LA)-LV pressure gradient during different phases of diastole. A higher E/A is predictive of increased LV filling pressure (which would impose greater diastolic wall stress, “preload”). E-wave velocity (LA-LV pressure gradient in early-diastole) is dependent on LV relaxation, whilst A-wave velocity (LA-LV pressure gradient during late-diastole) is dependent on LV stiffness and LA contractility (107). Kovacevic et al. found evidence for diastolic dysfunction (based on E/A ratios) in 61% (11/18) of their PA patient cohort, and diastolic dysfunction typically preceded the cardiomyopathy seen in the seven patients (7/18; 39%) in this study; four patients (4/18; 22%) had diastolic dysfunction in the absence of any LV systolic dysfunction.

Despite the observations in the study by Kovacevic et al. (106), prolongation of the QT interval is an established observation in PA (28, 33, 108). In a large multi-center study of 55 pediatric and adolescent PA patients from 16 European metabolic centers, 22% of patients (12/55) had a prolonged QTc (108). No ventricular arrhythmias were noted. Although, Grünert et al. comment that only 38/55 patients of their cohort had an EKG documented, and that in most cases, no stress-testing or Holter monitoring was performed.

Baumgartner et al. (33) found a higher incidence of prolonged QTc intervals in their longitudinal study of 10 PA patients (median age of 9 years). Patients underwent cardiology investigation including 12-lead EKG, 24-h Holter monitoring, exercise-tolerance test (where possible) and echocardiography. Prolonged QTc >440 ms was found in 70% (7/10) of patients, with a markedly prolonged QTc >460 ms in 60% (6/10) of patients. In this subset of patients, abnormal repolarization was evident in the form of pre-terminal T-wave inversion (which can be a sign of ventricular hypertrophy). Twenty percent (2/10) of these children were found to have arrhythmic events including sinus bradycardia, ventricular ectopic beats (ventricular premature beats) and couplets (salvos). LV function (LV FS) was reduced in 40% (4/10) of patients with prolonged QTc, and one patient had DCM. Intriguingly, no correlation was found between prolonged QTc and biochemical and metabolic indices (pH, ammonia, amino acids, acyl-carnitines, etc.).

Although the arrhythmias documented by Baumgartner et al. (33) were relatively innocuous, life-threatening arrhythmias in conjunction with QT prolongation have indeed been documented in PA. Jameson and Walter (109) reported successful resuscitation following sudden cardiac arrest in a 13-year-old girl who was diagnosed with PA shortly after birth whilst playing netball. Her echocardiogram was normal with no evidence of cardiomyopathy. However, 24-h Holster showed QTc prolongation of 450 ms, which was confirmed on exercise testing when QTc increased to ~500 ms during the recovery phase. The patient recovered well and was discharged with β-blockade therapy. However, cardiac arrest in association with cardiomyopathy in PA has also been described. Tan et al. (110) reported a 25-year-old man confirmed with PA at 2 years of age, who collapsed whilst jogging. Following cardiopulmonary resuscitation, paramedics identified VF which was successfully defibrillated. Initial investigation showed raised anion gap metabolic acidosis and lactic acidemia, but normal cardiac biomarkers and serum ammonia. QTc was normal (425 ms) and was unchanged from previous clinic EKGs as part of routine PA follow-up. However, post-arrest echocardiography showed LVEF to be reduced (35%), which was a further decrease from 3 months prior to cardiac arrest (49%).

### Liver Transplantation in the Treatment of PA-Induced Cardiomyopathy: Providing Clues to Underlying Mechanisms

Although PA treatment is not a focus of this *Review*, liver transplantation (LT) requires mentioning as it provides insights into the potential mechanisms of PA-induced cardiac dysfunction. For a more complete review of LT for the treatment of cardiac disease in PA, we refer readers to a recent study by Berry et al. (111). In brief, liver transplantation (LT) has been proposed to be an effective way to treat PA by Leonard (112). The liver is the major site for propionate metabolism (113). Since PCC is expressed in all tissues of the body, LT is not expected to be a metabolic cure, but rather to stabilize patients and minimize decompensations. Indeed, LT has been shown to be effective in improving clinical outcome in PA and to largely eliminate keto-lactic-acidosis crises (114, 115). Intriguingly, there have been several studies to suggest that LT can resolve cardiomyopathy in PA, beginning with the seminal report by Romano et al. (105), and indeed, LT is now considered a valid treatment for cardiac disease in PA. More recently, however, Berry et al. (111) reported a child whose cardiomyopathy had recurred following LT. This child was diagnosed with PA at 5 months and presented with signs of CHF at 9 years of age; subsequent echocardiogram found DCM with reduced LV EF (20%), and prolonged QTc (480 ms). These complications were successfully managed with inotropes and diuretics and cardiac function resolved. He received a successful LT at 10 years for other clinical indications. However, at 14 years of age he presented with cardiogenic shock and significantly elevated plasma propionyl-carnitine, 2-methylcitrate and glycine. He died at 18 years with pulmonary edema secondary to HF.

The pathogenesis of cardiomyopathy in PA is unclear. It appears to be unrelated to metabolic crises, since LT successfully reduces these episodes and improves overall outcomes. It should be noted, however, that propionate metabolites can remain elevated post-transplantation (111, 116). This leads to the credence that these “propionate metabolites” may be the key drivers of cardiac pathogenesis (insights into the mechanisms underlying cardiac dysfunction in PA from experimental animal models).

### Molecular Basis and Metabolic Signature of PA

PA occurs due to defective propionyl-CoA carboxylase (PCC), a biotin-dependent mitochondrial enzyme that normally catalyzes the carboxylation of propionyl-CoA to D-methylmalonyl-CoA, which eventually enters the Krebs cycle as succinyl-CoA (Figure 2). This is an important reaction since propionate is generated from various propiogenic substrates found in normal diets, including branched-chain amino acids (isoleucine, valine, methionine and threonine), odd-chain fatty acids and cholesterol (29).

PCC is encoded by *PCCA* and *PCCB* and missense, nonsense, and splicing mutations can occur in either of the genes (6). More than 150 variant alleles in each gene have been reported [(117), Human Gene Mutation Database Professional 2020.3 Release]. In both genes, missense mutations are predominant (~50%) and functional analyses have been used to identify functionally “null” mutations associated with the most severe phenotypes, as well as some hypomorphic alleles correlating with milder, late onset presentations (118, 119). However, most patients are compound heterozygotes, hindering the establishment of genotype-phenotype correlations in most cases (102, 120).

Regardless of which mutation is involved, the ensuing defect in PCC results in the accumulation of propionate and a myriad of its metabolites, including propionyl-CoA, propionyl-carnitine, 3-hydroxypropionate and 2-methylcitrate. It is clear that the accumulation of these substances is what is central to the pathophysiology of PA, for several reasons: (1) PA patients develop normally *in utero*, despite raised concentrations of propionate metabolites (e.g., propionic acid, 2-methylcitrate), and only develop symptoms following birth, as it is assumed that propionic acid crossing into the maternal circulation will be cleared by maternal hepatic PCC (121); (2) management of PA is centered around reducing the propionate-load, through dietary interventions and suppression of the gut flora; (3) although LT has largely shown success in managing PA as a “clinical syndrome,” propionate metabolites remain elevated post-transplant and recurrence of cardiomyopathy has been reported (see preceding Section); (4) despite being closely related biochemically, cardiac complications are far more common in PA compared to MMA, where there is less accumulation of propionate metabolites. Therefore, the question remains, which of these propionate metabolites are involved in the pathological remodeling of the heart, and why does it happen?

### Insights Into the Mechanisms Underlying Cardiac Dysfunction in PA From Experimental Animal Models

**Figure 3:**
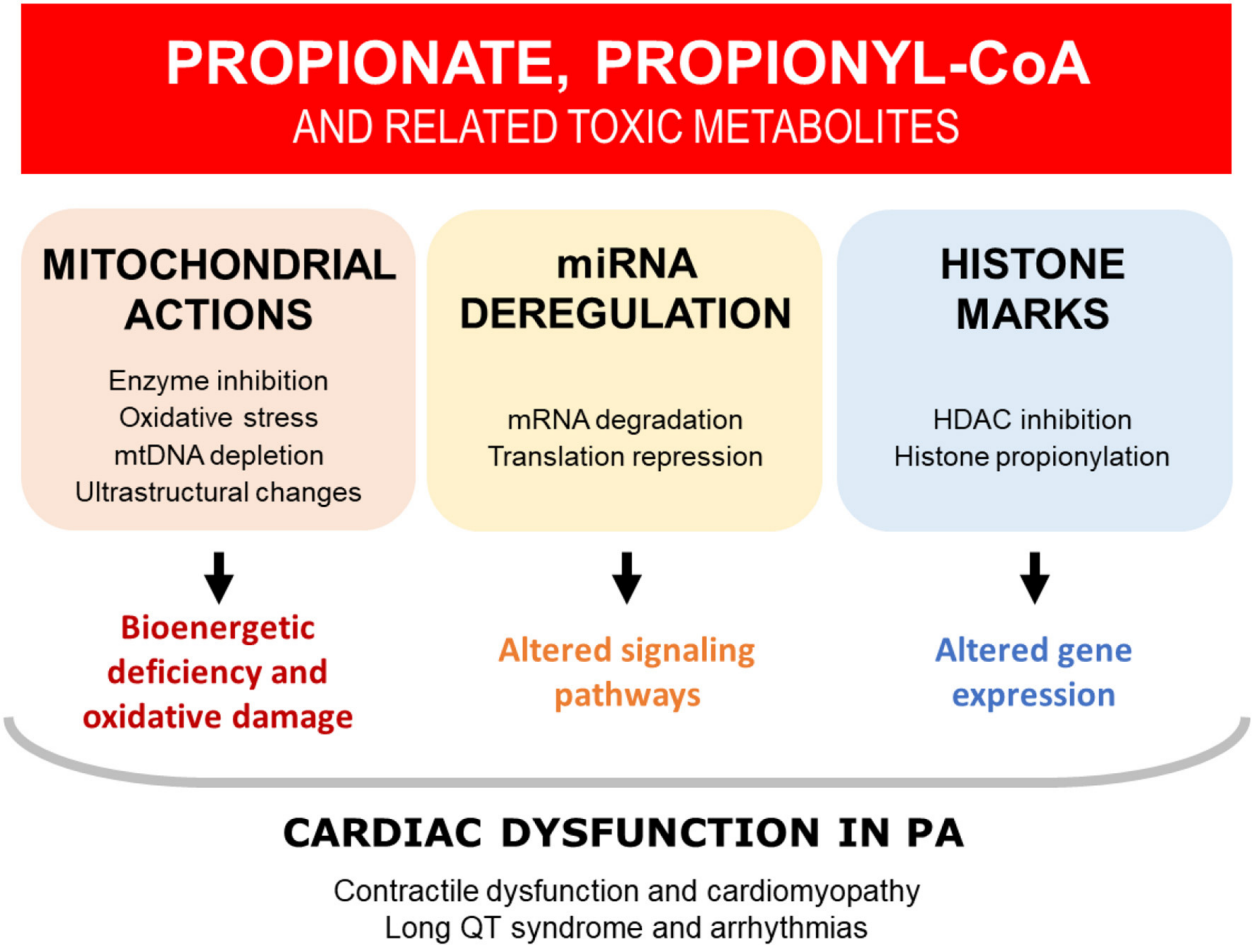
Overview of the potential mechanisms that may contribute to cardiac dysfunction in PA. The available evidence suggests that cardiac dysfunction in PA is driven by the toxic accumulation of metabolites, secondary to defective propionyl-CoA carboxylase (PCC). Multiple mechanisms have been identified, which are likely to be interrelated and acting in tandem. HDAC, histone deacetylase; miRNA, microRNA; mtDNA, mitochondrial DNA; PA, propionic acidemia.

PA is a complex, heterogeneous disease, affecting multiple organ systems and cellular pathways. It is therefore unlikely that a single mechanism is responsible for driving the heart disease in these patients (Figure 3). Whereas, the cardiomyopathy seems to appear mainly during childhood (mean onset age 7 years) (105), the long QT syndrome observed in PA has been shown to be progressive with age. The available evidence suggests that the DCM and prolonged QT are a secondary consequence of PCC dysfunction.

Here, we review the pathogenic mechanisms proposed to underpin cardiac dysfunction in PA. This knowledge-base has largely been borne from laboratory studies, exploiting a variety of experimental models. Broadly, two general “systems” have been used in studying cardiac dysfunction in PA: (1) *in vitro* systems, using patient-derived cells, immortalized cell lines, or primary rodent cells (which may or may not have been exogenously treated with propionate metabolites); (2) *Pcca*^−−/−^ (A138T) mice, an *in vivo* mouse model of PA generated by Dr. Michael Barry (Mayo Clinic, US). Since this mouse model has been utilized by numerous studies, it will be briefly described here.

*Pcca*^−/−^ (A138T) mice are estimated to have ~2% PCC activity as measured by a radiometric activity assay in liver homogenates (122). This residual level of activity results in the necessary minimum clearance of propiogenic substrates to allow these animals to survive to adulthood. Guenzel et al. (122) found these animals to present with a mild-to-moderate PA phenotype and have raised plasma markers characteristic of PA such as propionyl-carnitine and 2-methylcitrate. Furthermore, these animals have raised *Nppb* in their hearts, which encodes for the hormone brain-type natriuretic peptide (BNP), a classical biomarker released during hemodynamic stress (when ventricles are dilated, hypertrophic, or subjected to increased wall tension) (123).

#### Mitochondrial Dysfunction: Energetic Deficiency, Oxidative Stress, and Metabolic Dysfunction

The heart is a highly energy-demanding organ, with an average adult human heart consuming ~6 kg of ATP per day, despite only having enough stored for a few heartbeats. Thus, perhaps not surprisingly, mitochondrial dysfunction has been implicated as a major driver of pathological cardiac remodeling in cardiomyopathy (124). Although decreased energy supply is considered as the main consequence of mitochondrial dysfunction, recent advances have shown that it contributes to the pathogenesis of HF, beyond the idea of simply inadequate energy provision (125). Available evidence suggests mitochondrial dysfunction is an underlying etiological factor contributing to cardiomyopathy in PA in the form of ultrastructural changes, electron transport chain (ETC) deficiency, oxidative stress, and metabolic dysfunction (6, 102).

The accumulation of propionate metabolites can impair or interfere with mitochondrial biochemical pathways. Propionyl-CoA has been shown *in vitro* to be a major mitochondrial toxin in PA and to impair oxidative phosphorylation through the synergistic inhibition of pyruvate dehydrogenase complex, α-ketoglutarate dehydrogenase complex, and ETC Complex III (126). Deficiencies in ETC Complex I (103) and ETC Complexes III/IV (127) have also been detected in the hearts of PA patients. Baruteau et al. (128) presented a PA patient with severe DCM who was found to have enlarged mitochondria, atypical cristae, reduced Complex IV activity, as well as markedly reduced coenzyme Q_10_ (ubiquinone) on myocardial biopsy. Interestingly, the DCM improved with ubiquinone supplementation, which may be related to positive effects from reducing oxidative stress (see next).

There is a wealth of literature supporting that oxidative stress has a detrimental effect on the heart, including pathological remodeling, fibrosis, and contractile dysfunction (129). The redox state in the heart is balanced by sources of reactive oxygen species (e.g., mitochondrial electron transport, NADPH oxidases, uncoupled nitric oxide synthase) and intrinsic antioxidants/antioxidant systems (e.g., ubiquinone, vitamin C, superoxide dismutase) (129). Since oxidative phosphorylation is inhibited in PA, a redox imbalance and increased oxidative stress are predicted for PA hearts. Indeed, oxidative stress has been implicated as an important mechanism involved in the pathophysiology of PA (30), which is likely to be involved in PA-induced cardiac dysfunction. Increased intracellular hydrogen peroxide has been detected in PA patient fibroblasts, indicating increased oxidative stress is apparent in PA (130). In experimental studies, increased superoxide production has been shown in the hearts of *Pcca*^−/−^ (A138T) mice (131). In a subsequent study, treatment of *Pcca*^−/−^ (A138T) mice with antioxidants was found to normalize the increase in expression of *Nppb* mRNA (BNP) in the heart (132). The relevance of oxidative stress as a mechanism contributing to cardiomyopathy is supported by the clinical experience of ubiquinone supplementation improving DCM, as described in the previous paragraph.

The heart relies on FAO as its primary substrate for ATP production. However, it also displays a remarkable ability to utilize different substrates, including ketone bodies and amino acids, with a so-called “metabolic flexibility” (133). Despite this flexibility, a hallmark of pathological hypertrophy and HF is a “switch” in substrate-dependence from fatty acids toward glucose and ketone bodies (124, 134). Using an isotope-based metabolic flux approach in Langendorff-perfused rat hearts, Wang et al. (135) found that the perfusion of propionate led to an accumulation of propionyl-CoA. This accumulation caused significant mitochondrial CoA trapping and altered pool sizes of Krebs cycle intermediates. FAO was inhibited, with a concomitant increase in glucose oxidation (despite glycolysis being unchanged). Interestingly, supplementing the hearts with L-carnitine did not relieve the CoA trapping or reverse the fuel-switch mediated by propionate, because the authors showed that the heart lacks the specific enzyme that catalyzes this reaction. Thus, there is good evidence for metabolic dysfunction contributing toward cardiac dysfunction in PA. Furthermore, this hindering of cardiac energy metabolism is likely to be exacerbated by 2-methylcitrate, a commonly elevated metabolite in PA, formed from propionyl-CoA, which has been shown to act as an inhibitor of Krebs cycle enzymes (136).

#### MicroRNAs

Whilst only 3% of the human genome codes for proteins, the remainder is still extensively transcribed, producing long non-coding RNAs (>200 nucleotides) and microRNAs (miRNAs; ~22 nucleotides), which are recognized to play important roles in transcriptional regulation (137). miRNAs are short, non-coding, single-stranded RNAs that anneal with complementary mRNA sequences to suppress protein expression. There are a plethora of miRNAs that have been discovered which have been implicated as key players in the pathophysiology of heart disease, and are uniquely detected in different cardiac pathologies like arrhythmia, cardiac hypertrophy and heart failure (138). For example, Wahlquist et al. (139) showed 15 miRNAs to be uniquely upregulated in a mouse model of HF. Interestingly, miRNA-25 was found to reduce expression of SERCA, a key cardiac protein important in excitation-contraction coupling (see excitation-contraction coupling). Perhaps more importantly, anti-miRNA-25 therapy was found to restore cardiac function in these failing mice, along with reduction of fibrosis and normalization of cardiomyocyte size.

Dysregulated miRNAs have also been detected in a range of tissues (e.g., brain, liver) in *Pcca*^−/−^ (A138T) mice (140). More recently, Fulgencio-Covian et al. (141) performed analysis of cardiac-enriched miRNAs in a cohort of PA patient plasma and in two experimental models: (1) the hearts of *Pcca*^−/−^ (A138T) mice, and (2) propionate treatment in HL-1 cells, an immortalized atrial cell line. The authors found a significant upregulation of several miRNAs involved in cardiac pathology in *Pcca*^−/−^ (A138T) mouse hearts, for example miRNA-22 which has been implicated in cardiac hypertrophy and fibrosis that acts via the PI3K/AKT pathway. To support the relevance of these findings, Fulgencio-Covian et al. found upregulation of several genes involved in the PI3K/AKT pathway that are important for the heart (e.g., *Fos, Jun*). Finally, experiments were performed on HL-1 cells treated with propionate, which confirmed the upregulation of several miRNAs increased in PA mice, providing evidence that propionate is a toxic metabolite in PA.

#### Histone Acetylation and Propionylation

DCM is frequently inherited through mutations in the “classical DCM genes” such as *TTN* (titin) and *LMNA* (lamin A/C) (142). Although the evidence to-date shows that cardiomyopathy in PA can occur without a change in expression of the DCM genes, the possibility exists that PA acts as a modifier of an “unknown” DCM-related gene, or of genes critical to normal cardiac function (143). Post-translational modification of histone proteins, in the form of acylation, is a major mechanism of regulating gene expression, and epigenetic alternations in cardiac gene expression programs are known to be involved in adverse cardiac remodeling (144). It is established that acetyl-CoA is not the only metabolite that can participate in this process, but rather a plethora of metabolites such as butyryl-CoA, succinyl-CoA and notably, propionyl-CoA (145).

There are two pathways implicated as potential mechanisms in the epigenetic regulation of gene expression in PA: (1) the inhibition of histone deacetylases (HDACs) by propionate, promoting histone acetylation, which is known to be involved in pathological cardiac remodeling (146); (2) propionyl-CoA acting as a false-substrate for histone acetyltransferases (HATs) to result in histone propionylation. Butyrate (C_4_) is a well-established, potent inhibitor of HDACs, and propionate (C_3_) has shown to also have HDAC inhibition properties (147). It would be expected that the accumulation of propionate in PA would result in HDAC inhibition and an increase in histone acetylation. Histone 3 propionylation at lysine 14 (H3K14pr) has recently shown to be a modification that is a signature of transcriptionally active chromatin. The study by Kebede et al. (148) showed that H3K14pr is present in the liver of *Pcca*^−/−^ (A138T) mice and that it is mediated *in vivo* by HATs (GCN5 and PCAF). They also showed that H3K14pr levels return to baseline wild-type levels in those mice that underwent *PCCA* gene therapy.

#### Ion Channel Function and the Cardiac Action Potential

Prolongation of the QT interval, as seen in long QT-syndrome (LQTS), is a major pro-arrhythmic substrate that can precipitate in sudden cardiac death due to VF and torsades de pointes (149). LQTS can be both acquired (e.g., drug-induced, cardiomyopathy-induced) and inherited (due to mutations in genes, e.g., *KCNQ1, KCNH2*) (150). Thus, whilst the small possibility exists that PA patients carry mutations in genes encoding for *both* PCC (i.e., PA) and ion channel subunits (i.e., congenital LQTS), this has never been reported, and indeed, whole-exome sequencing is frequently employed during IMD diagnosis (2, 119). Furthermore, as Baumgartner et al. (33) pointed out, mutations in ion channel subunits are unlikely to be the cause of LQTS in PA patients, since QT prolongation was not permanently present in PA, and because prolongation of the QT appeared to worsen with age as a progressive process (vs. congenital LQTS, which typically presents at birth) (see first observations and typical clinical features of heart disease in PA). Therefore, LQTS in PA is most likely to be the acquired form, due to cardiomyopathy, and/or, the toxic effect of propionate metabolites. Although, it should be noted that Baumgartner et al. (33) found no correlation between prolonged QTc and biochemical and metabolic indices (pH, ammonia, amino acids, acyl-carnitines, etc.).

QT intervals recorded on surface EKGs are ultimately a manifestation of the ventricular action potential (AP). Since LQTS can be caused by a defect in a variety of currents underlying the AP (primarily *I*_Kr_ and *I*_Ks_), direct examination of these currents by electrophysiology is required to identify the underlying ionic mechanism (and therapeutic target). Bodi et al. (151) interrogated the pathophysiology of LQTS in PA using electrophysiology. Their study investigated the acute and chronic effects of various propionate metabolites (propionic acid, propionyl-carnitine and 2-methylcitrate) on *I*_Kr_ (*KCNH2*) and *I*_Ks_ (*KCNQ1*), the most important repolarizing currents. Although *I*_Kr_ gating properties were found to be affected by propionate metabolites, the main effects were found to be on *I*_Ks_. Acute application of propionate metabolites significantly decreased *I*_Ks_ current densities (tail and end-pulse currents), and these effects were reversible (upon washout). The effect of a chronic application of propionate metabolites on protein expression found KCNQ1 to be reduced, but only by propionyl-carnitine and 2-methylcitrate (propionic acid paradoxically increased KCNQ1 expression, although this had no effect on *I*_Ks_ current density). Finally, propionic acid significantly prolonged AP duration (APD) in human-induced pluripotent stem cell-derived cardiomyocytes, but not in KCNQ1-deficient rabbits lacking *I*_Ks_. Collectively, their results suggested a reduction of *I*_Ks_ may account for the observed APD prolongation and QT interval prolongation in PA.

#### Excitation-Contraction Coupling

Cardiac excitation-contraction (E-C) coupling is the process by which mechanical contraction is triggered by an electric stimulus. Cardiomyocyte Ca^2+^ handling and E-C coupling frequently becomes dysregulated in a number cardiac pathologies, including cardiomyopathy and arrhythmia (152, 153). Following an AP, depolarization of the myocyte causes Ca^2+^ entry via voltage-gated L-type Ca^2+^ channels as an inward current (*I*_Ca, L_). This Ca^2+^ entry triggers a much larger release of Ca^2+^ from the myocyte's intracellular Ca^2+^ store, the sarcoplasmic reticulum (SR), via ryanodine receptor release channels in a process termed Ca^2+^-induced Ca^2+^-release (154). Contraction is triggered by the rise in free intracellular [Ca^2+^], causing Ca^2+^ to bind to troponin C on the myofilament and promoting cross-bridge formation (88). Relaxation occurs through removal of Ca^2+^ from the cytoplasm through a combination of (1) Ca^2+^ being pumped back into the SR by the SR-Ca^2+^-ATPase (SERCA), and (2) Ca^2+^ extrusion out the cell by Na^+^/Ca^2+^-exchange (NCX) (153).

Identifying which E-C coupling protein is defective for a given pathology is important for therapeutic strategies. Only one study has been published to-date interrogating E-C coupling in PA. Tamayo et al. (7) performed Ca^2+^ imaging on ventricular myocytes isolated from *Pcca*^−/−^ (A138T) mice. Compared to wild-type controls, they found electrically-evoked Ca^2+^ transients were smaller, and took longer to recover, indicative of reduced SERCA activity. NCX activity was found to be unchanged (probed from the caffeine-evoked Ca^2+^ transient). Although SERCA protein expression was unchanged, SERCA pump activity (assayed by ATP hydrolysis) was reduced, which the authors attributed to reduced energy metabolism (that is typical in PA), and oxidation of SERCA (secondary to increased ROS in these myocytes). Since reduced SERCA function is a hallmark of cardiomyopathy (152) and aberrant Ca^2+^ handling increases the propensities for arrhythmias (155), remodeled E-C coupling could be another mechanism contributing to adverse cardiac function in PA.

## Conclusions

Genotypically, organic acidemias could be considered as “simple” enzymatic lesions, but their phenotypic presentation can be very complex, highlighting the breadth of actions of the metabolites associated with the defect. Cardiac dysfunction is a common complication in several OAs, including Barth syndrome and propionic acidemia. However, the mechanistic basis underlying this dysfunction has yet to be fully elucidated, both owing to the complexity and rarity of these diseases.

There has been a growing interest in the biology of propionate and in the research effort to understand the mechanisms underlying gross organ dysfunction in PA, particularly in the past decade. Although it is theoretically possible that PA patients have congenital defects in PCC and in proteins that are typically mutated in DCM or LQTS, this is highly unlikely. Rather, available evidence suggests that these cardiac pathologies are driven by the accumulation of toxic metabolites, including propionate, propionyl-CoA and 2-methylcitrate. There are multiple mechanisms that have been identified, including mitochondrial dysfunction, oxidative stress and changes in gene expression. However, it is unlikely that there is a single mechanism acting in isolation. A greater understanding of these processes and identifying the molecular targets in the hearts of PA patients will help in formulating better therapeutic strategies for the future.

## Author Contributions

All authors listed have made a substantial, direct and intellectual contribution to the work, and approved it for publication.

## Conflict of Interest

The authors declare that the research was conducted in the absence of any commercial or financial relationships that could be construed as a potential conflict of interest.
